# A Spectacular Northeast Pacific Invasion by a Low Genetic Diversity Parasite, *Orthione Griffenis*

**DOI:** 10.1002/ece3.71160

**Published:** 2025-03-23

**Authors:** Emily R. Curcio, Viridiana Avila‐Magaña, Joshua Mayo, Leanne E. Elder, Kelly R. Martin, Grace K. Roa, John W. Chapman, Jingchun Li

**Affiliations:** ^1^ Department of Ecology and Evolutionary Biology University of Colorado Boulder Boulder Colorado USA; ^2^ Department of Ecology and Evolutionary Biology University of California Santa Cruz Santa Cruz California USA; ^3^ Museum of Natural History University of Colorado Boulder Boulder Colorado USA; ^4^ Department of Fisheries, Wildlife and Conservation Hatfield Marine Science Center Newport Oregon USA

## Abstract

Invasive marine parasites can be established long before their introduction mechanisms are resolved, and factors contributing to their successes are often unknown. Understanding the ecology of these invasive parasites is urgently needed for economic and resource conservation efforts. In the eastern Pacific, the introduced Asian bopyid parasite, *Orthione griffenis*, extends at least from Sitka, Alaska, USA to San Quintín, Baja California, Mexico. In the new range, it infests at least two native hosts and one introduced host. We examined the genetic structure of *O. griffenis* from Morro Bay, California, to Ketchikan, Alaska, based on Double digest restriction‐site associated DNA (ddRAD) sequencing, and estimated its larval dispersal range from laboratory‐based survival tests. There was a lack of genetic diversity, structure, and isolation by distance across *O. griffenis* populations. There was also a lower‐than‐expected genetic polymorphism, consistent with previous hypotheses of its dispersal away from a single colonization event by a small number of initial propagules. *Orthione griffenis* larval survival appears sufficient for dispersal in coastal ocean currents over the observed northern invasion range and for transpacific dispersal via ballast water. The natural history and interaction of *O. griffenis* with its new and original hosts provide a unique system for understanding species adaptation in invaded ecosystems. This work demonstrates how genetically homogeneous invasive parasite populations can rapidly expand and potentially alter marine communities. Expanded efforts to understand the interactions of parasites and their vectors in their native and non‐indigenous habitats are critically needed for detecting, limiting, and mitigating their effects on endemic marine communities.

## Graphical abstract

This study examined the genetic structure of the invasive isopod parasite, *Orthione griffenis*, between Morro Bay, California, and Ketchikan, Alaska, using ddRAD sequencing and estimated its larval dispersal range from laboratory‐based survival tests. There was a lack of genetic diversity, structure, and isolation by distance across *O*. *griffenis* populations. This work highlights the potential for genetically homogeneous invasive parasite populations to rapidly expand and force fundamental alterations of marine communities.

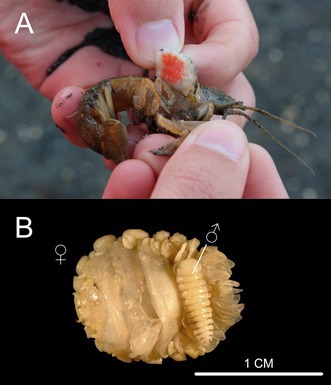

## 1 Introduction

Invasive species threaten the world's ecological and economic well‐being (McNeely et al. 2001; Diagne et al. 2021) by reducing biodiversity, altering organism interactions, and eradicating native species (Kumschick et al. 2015; Bellard et al. 2016; Goedknegt et al. 2016). Biological invasion research has emphasized terrestrial habitats (Lowry et al. 2013; Jeschke et al. 2014), but marine invasions have been rapidly increasing due to global increases in shipping, aquaculture, and live seafood trade (Bax et al. 2003; Molnar et al. 2008; Grosholz 2002). Research on introduced marine parasites has, relative to other taxa, remained particularly meager (Ruiz et al. 2000), with less than 2% of invasive biology research focused on parasitic interactions (Poulin 2017). Most aquatic parasites are small and have complex life cycles that include multiple host species (Parker et al. 2003; Auld and Tinsley 2014) and can be easily overlooked and unexamined in standard biodiversity surveys (Chapman et al. 2021). Parasites can, nevertheless, alter marine ecosystems and food webs (Lafferty et al. 2008), oftentimes by altering host reproduction (Lafferty and Kuris 2009) and behaviors (Mouritsen and Poulin 2003). Successful marine conservation strategies thus require increasingly detailed information on introduced marine parasites.

**FIGURE 1:**
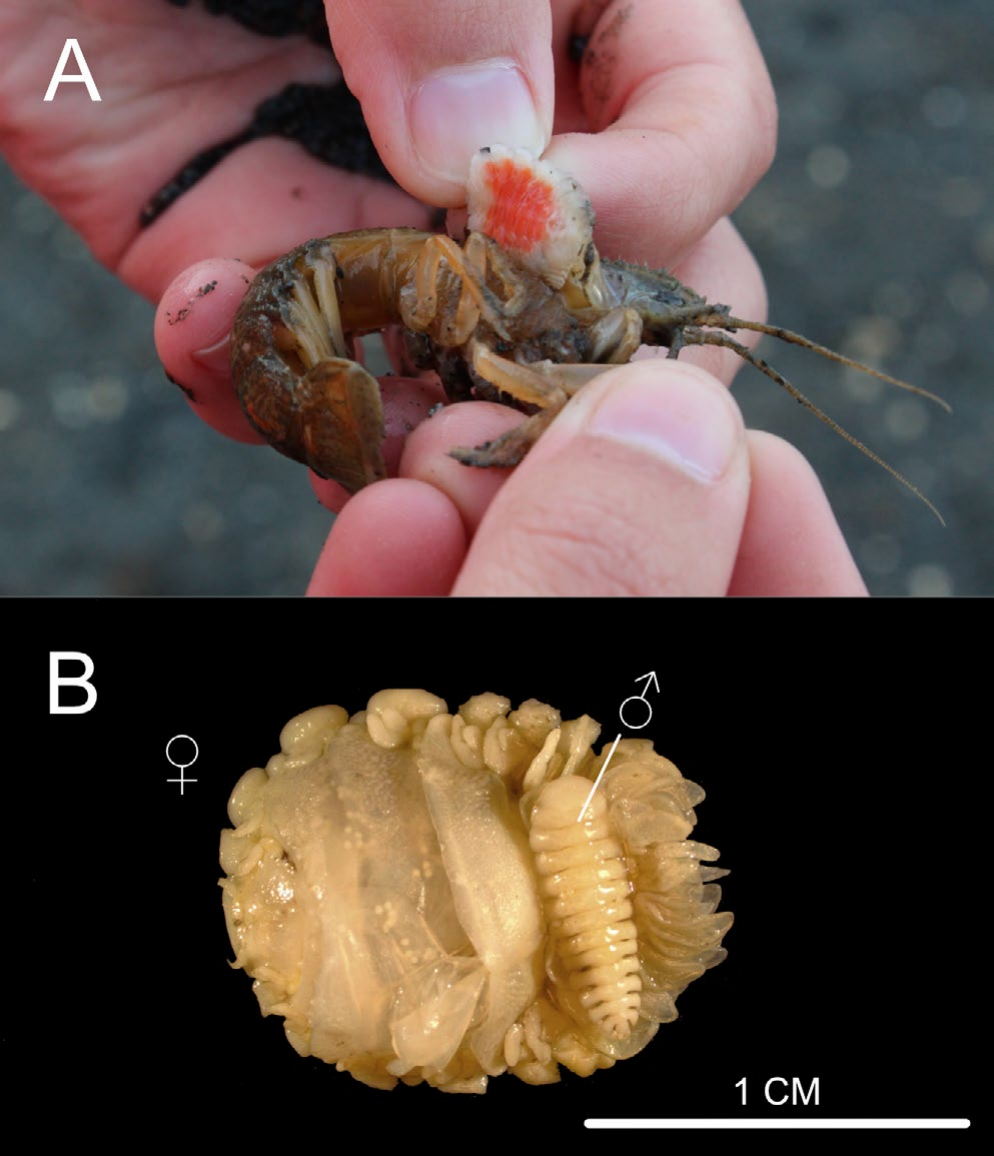
(a) An infected *Upogebia pugettensis* with a mature female *Orthione griffenis* exposed from under the host carapace. (b) *Orthione griffenis* female and male (UCM 51079). Photos: E. Curcio.

Marine estuaries are among the world's most invaded ecosystems (Cohen and Carlton 1998). One crucial aspect of estuary function is bioturbation, the redistribution and reworking of sediments due to animal activities (Richter 1936; Lohrer et al. 2004; Shull 2009). The blue mud shrimp, *Upogebia pugettensis* (Dana 1852), native to the Pacific Coast of North America (Figure 1), are among the most abundant bioturbators in Northeast Pacific estuaries. Their extensive burrow galleries increase estuary benthic surface areas that help cycle nutrients (D'Andrea and DeWitt 2009) and create habitats for many obligate and facultative commensal species (Chapman et al. 2012; Li and Ó Foighil 2012). *Upogebia pugettensis* is the only native mud shrimp north of Morro Bay, California (Williams 1986), and reaches Prince William Sound, Alaska.

**FIGURE 2:**
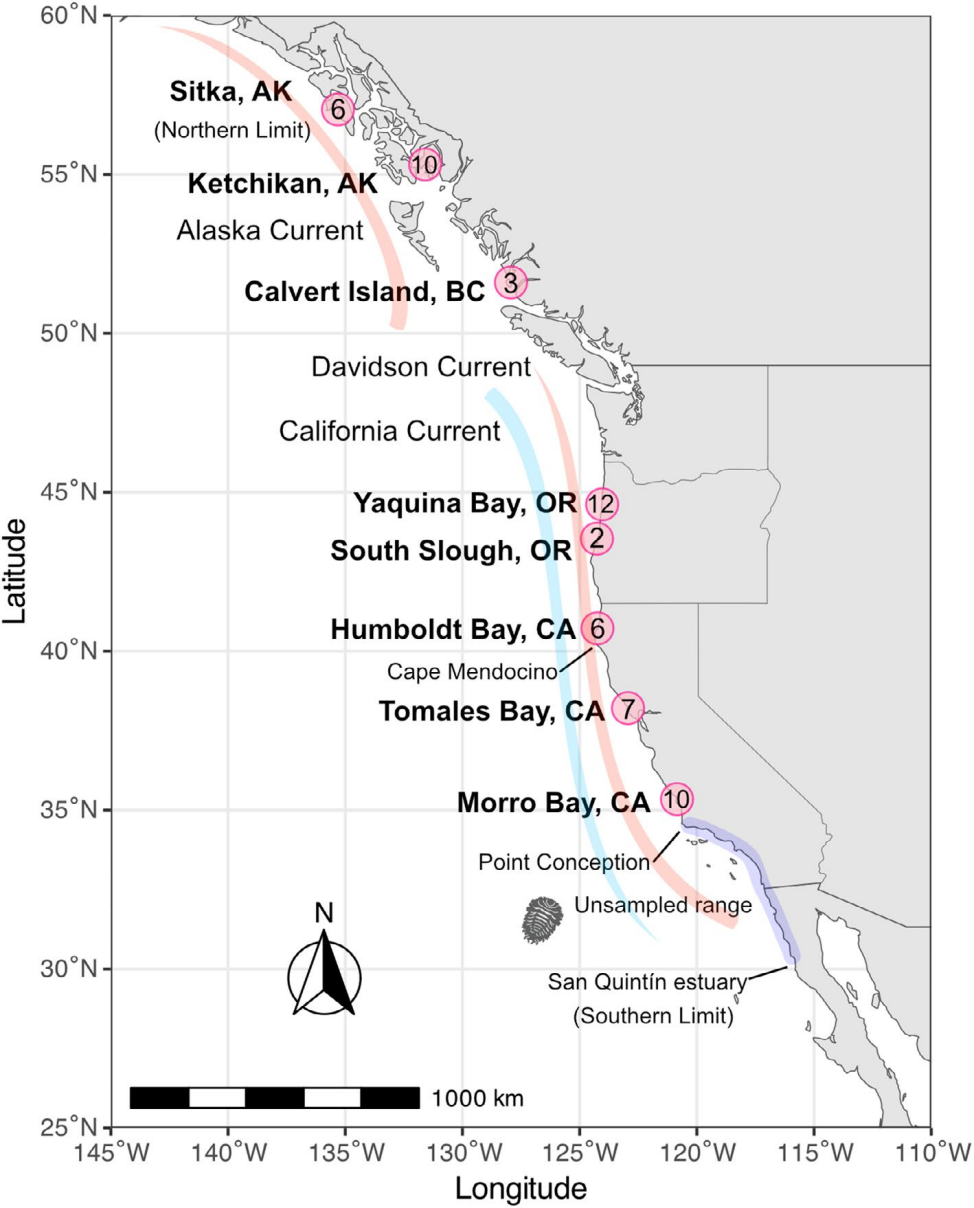
*Orthione griffenis* sampling locations. Red dots indicate all locations that have been surveyed/sampled and their corresponding sample sizes. The shaded area represents the unsampled range in this study.

The largest parasitic bopyrid isopod in North America, *Orthione griffenis* (Markham 2004), was introduced to the Northeast Pacific from Asia in the 1980s (Williams and An 2009; Chapman et al. 2012) (Figure 1) and infests *U. pugettensis* . *Orthione griffenis* attaches to the inside of the *U. pugettensis* carapace lining and feeds on the host's hemolymph. Castration of female *U. pugettensis* due to hemolymph losses is consistently documented (Smith et al. 2008; Griffen 2009; Repetto and Griffen 2011; Asson et al. 2017). The lost fecundity is closely associated with catastrophic *U. pugettensis* population declines over the species' range (Dumbauld et al. 2011; Chapman et al. 2012, 2021; Chapman and Carter 2014). The presently known *O. griffenis* range (Figure 2) extends over 3000 km and 25° latitude, from San Quintín estuary, Mexico, to Sitka, Alaska (Campos et al. 2010; Chapman et al. 2021) and six biogeographical provinces (Blanchette et al. 2008). All *U. pugettensis* populations examined thus far, from its southern range limit Morro Bay, California, to Sitka, Alaska, are infested, and we are unaware of uninfested *U. pugettensis* populations anywhere (Chapman et al. 2012, 2021; Chapman and Carter 2014). Addressing the collapse of *U. pugettensis* populations and conserving northeastern Pacific estuary communities requires a deeper understanding of host–parasite interactions and the mechanisms driving the establishment and spread of *O. griffenis* along the West Coast of North America.

Bopyrid isopods disperse as larvae. Information on parasite larval survival time in the open ocean (an indicator of dispersal potential) and genetic diversity (an indicator of connectivity) can reveal invasion histories and host–parasite interactions. If *O. griffenis* arrived in the early 1980s via ballast water from cargo ships, its larval half‐life would need to exceed the transit time of a transpacific voyage. Modern transportation data (www.fluentcargo.com) indicate that the typical transit time between major Asian ports and the U.S. West Coast is approximately 17 days. Given advancements in ship technology and logistics over the past few decades, transit times in the 1980s were likely longer—probably exceeding 20 days. However, no data are currently available on *O. griffenis* larval survival time to assess the feasibility of its transpacific dispersal.

*Orthione griffenis* larval stages begin with 0.3 mm length free‐swimming, non‐feeding epicarids that hatch from the female bopyid's brood pouch and emigrate from the estuary on ebb tides into the coastal ocean waters (Chapman et al. 2012). The epicarids settle onto pelagic copepod hosts (Chapman et al. 2012) where they morph into a parasitic microniscus stage (Saito 2002; Chapman et al. 2012). All Asian and North American microniscan records (Saito 2002; Chapman et al. 2012) are from coastal marine calanoid copepod hosts. The microniscans grow to approximately 0.7 mm and morph into a non‐feeding, free‐swimming cryptoniscus stage, then return to the estuary and settle onto *Upogebia* hosts where they morph into the final parasitic, reproductive bopyridan stage (Chapman et al. 2012; Baeza 2015). The epicaridan and microniscan life stages are likely capable of surviving for many days or weeks (Baeza 2015), but they are difficult to collect in the open ocean and maintain in laboratory cultures. In contrast, the subsequent non‐feeding cryptoniscus stage, which occurs in estuaries, allows for a conservative estimate of pelagic larval dispersal (PLD) based on their survival in static seawater cultures. Additionally, newly settled cryptoniscans enable taxonomic differentiation from the only other common cryptoniscus species in northeastern Pacific estuaries, *Ione cornuta* (Bate, 1865), minimizing the risk of misidentification (Chapman et al. 2012). Finally, controlled laboratory conditions provide an opportunity to assess whether abiotic factors such as temperature and light significantly affect cryptoniscus survival.

Our goal was to better understand the dispersal potential of *O. griffenis* and its post‐invasion biogeography. Chapman et al. (2021) proposed that North American *O. griffenis* was established and rapidly spread from a single founding population. If this is true, we expect extended pelagic larval survival in the open ocean and low genetic variation across its geographical range. Alternatively, the current wide distribution of *O. griffenis* could result from multiple introductions of diverse Asian populations over time. If so, we expect high genetic diversity, significant genetic structure, and restricted larval survival relative to ocean circulation. Additionally, limited genetic assessments of *U. pugettensis* host populations suggest that Cape Mendocino, CA, may serve as a genetic barrier, with northern and southern populations exhibiting moderate genetic differentiation (Horan 2021). While host and parasite population structures do not always align—particularly in the case of an invasive parasite and a native host—abiotic factors such as ocean currents that drive host population divergence may similarly influence parasite populations. Therefore, it is worth investigating whether *O. griffenis* exhibits a comparable genetic breakpoint to *U. pugettensis* .

In this study, we first used Double digest restriction‐site associated DNA (ddRAD) sequencing (Peterson et al. 2012) to assess *O*. *griffenis* population genetic structure over its California to Alaska range that is presently invaded. We also assessed the potential effects of isolation by distance and geographic barriers on *O. griffenis* population structure. Additionally, the survival times of *O. griffenis* larvae in ballast water, other human dispersal vectors, or nearshore coastal currents have never been examined. We therefore examined average durations of cryptoniscan survival in lab settings to infer their probable dispersal distance.

## 2 Methods

### 2.1 Specimen Collection for Population Genetic Analyses

We collected *Orthione griffenis* adults for genetic analyses from eight locations ranging from Morro Bay, California, to Sitka, Alaska (Figure 2). Shrimp hosts were extracted from their burrows using hand tools, which included yabby pumps (a piston‐like suction device that extracts shrimp from their burrows), shovels, and/or hand cores. Extracted sediments were washed over a 3 mm mesh sieve to locate shrimps. Every host was visually inspected to search for and collect *O. griffenis* (if they were infected). A subset of the collected *O. griffenis* specimens was frozen and shipped to Boulder, Colorado, to be preserved in 95% EtOH on arrival; others were placed directly in 95% EtOH. The specimens are deposited at the University of Colorado Museum of Natural History (UCM) (Table S1). An additional five *O. griffenis* (from Calvert Island, Canada) were loaned from the Florida Museum of Natural History to add another population to this study.

### 2.2 Genetic Sequencing and Processing

We used ddRAD sequencing (Peterson et al. 2012) for genome‐wide single nucleotide polymorphism (SNP) detection in *O. griffenis* (Lal et al. 2015). For female *O. griffenis*, which are larger, tissue was extracted from the abdomen. For male specimens, the posterior half of the body or the entire specimen was used for extraction. The Qiagen Blood and Tissue kit (Germantown, MD) was used to extract genomic DNA from *O. griffenis*. DNA concentration and quality were assessed using the QUBIT fluorometer (Invitrogen) and agarose gel electrophoresis. Samples with a concentration higher than 10 ng/uL and with clear bands from gel electrophoresis were sent for subsequent library preparation and sequencing.

We prepared ddRAD libraries using the Peterson et al. (2012) protocol. DNA was digested using the enzymes PstI and MseI, and then ligated with adaptors to facilitate multiplexing. Fragmented DNA was pooled and purified using magnetic beads, and the purified fragment sizes were selected using the Pippin Prep system (Sage Science). The selected fragments were amplified using PCR to increase the sequencing library concentration and to add annealing sequences and multiplexing indices. Libraries were sequenced using Illumina NovaSeq (100 bp single‐end) to create an average sequencing depth of 25×. Sequencing was performed by Floragenex (Portland, OR) and all sequence data were deposited in NCBI Genbank (Curcio 2024).

Raw sequencing data were demultiplexed, quality‐controlled, and trimmed using STACKS (Andrews 2010; Catchen et al. 2013) and the sequenced data from individual *O. griffenis* were pooled for *de novo* locus assembly using the denovo_map.pl script in STACKS. We used the recommended r80 method in STACKS de novo to maximize the number of polymorphic loci (Catchen et al. 2013; Paris et al. 2017). We created output files using the–write‐single‐snp flag to ensure there is only one SNP per RAD locus.

The dataset with completed SNP calling was filtered using the max‐missing flag in VCFtools (Danecek et al. 2011) to exclude sites with more than 20% missing data. The filtered variant call format (vcf) sample files were reordered to verify individual metadata were associated with their corresponding SNPs using BCFtools (Li and Barrett 2011). Samples with a read depth below 10× were removed.

### 2.3 Population Genetic Analysis

The final dataset after filtering included 56 individuals from 8 locations, from California to Alaska. Eleven of these individuals were male, and 45 were female.

General genetic diversity parameters including observed heterozygosity (H_O_), expected heterozygosity, within‐population gene diversity (H_S_), overall gene diversity (H_T_), and Tajima's D (assessing genetic polymorphisms) were calculated to provide an overall examination of *O. griffenis'* genetic variation. Pairwise fixation index and overall fixation index (F_ST_) were used to examine divergence among populations (Weir and Cockerham 1984). The inbreeding coefficient (F_IS_) was also calculated. All parameters were calculated in R 1.3.1093 (R Core Team 2020) using the packages {adegenet} (Jombart and Ahmed 2011), {hierfstat} (Goudet 2005), and {stamp} (Pembleton et al. 2013). Per‐SNP F_ST_ and outlier loci were assessed using the R package {OutFLANK v 0.2} (Whitlock and Lotterhos 2015).

Principal Component Analysis (PCA) was performed using the R package {SNPRelate} (Zheng et al. 2012) in three runs to visually assess potential clustering among populations based on overall SNP composition. Each analysis was performed after removing visually obvious outliers from previous PCA runs. A TESS analysis was performed using the R package {tess3r} (Caye et al. 2016) to characterize the population assignment of sampled individuals based on SNP data. We used cross‐validation to determine the most likely *K*‐value, or number of ancestral contributions, and visualized the population assignment results using bar plots of estimated ancestry proportions using the R package {tess3r}.

The relationships between *O*. *griffenis* population geographic distances and genetic differences were assessed using a general linear model (R package {stats}). Geographic distance among populations was estimated using the NOAA Latitude/Longitude Distance Calculator (https://www.nhc.noaa.gov/gccalc.shtml) and pairwise F_ST_ values were used for genetic differences.

We used Analysis of Molecular Variance (AMOVA) to assess the genetic structure of *O. griffenis* along the West Coast. This helps evaluate degrees of genetic variation within individuals, between individuals within populations, between populations, and among clusters of populations in northern and southern regions. Cape Mendocino, CA, is a suspected location of population differentiation among *U. pugettensis* (Horan 2021). Based on this information, Cape Mendocino was used to define northern and southern regions. AMOVA was performed using the R package {pegas} (Paradis 2010).

### 2.4 Average Cryptoniscan Survival Times

We tested whether average *O. griffenis* cryptoniscan larval survival times are sufficient for trans‐Pacific crossings in ballast water traffic and/or for long‐range passive dispersal in seasonal coastal currents of western North America (Chapman et al. 2012, 2021) by experimental measures of larval mortality rates.

Our survival tests included a 58‐day experiment conducted between 6/22/2021 and 8/19/2021 and a 17‐day experiment conducted between 8/2/2021 and 8/19/2021. The 58‐day experiment consisted of replicated treatments of constant dark at 12°C, constant dark at 20°C, and indirect day and night (diel) light at 20°C. These variables represent environmental conditions that the larvae may experience during natural dispersal on currents. The temperature selection was based on records of eastern Pacific coastal ocean temperatures ranging from ~12°C (Behrens Yamada et al. 2021) to at least 18°C (surf‐forecast.com). The diel light cycle was included based on known vertical migration patterns of *O. griffenis* cryptoniscans in Yaquina Bay (Chapman, personal observations). The 58‐day experiment was terminated because it was sufficiently long to estimate the half‐life of most individuals, and we did not mean to estimate maximum survival time.

The 17‐day experiment was designed to test for larval survival in unexchanged ballast water conditions. This particular timeframe was chosen because cargo ship ocean passages from Yokohama, Japan, in the western Pacific range of *O. griffenis* (Chapman et al. 2012), to Tacoma, Washington, are approximately 17 days (www.fluentcargo.com).

We collected cryptoniscans for these experiments in a 150 μ mesh plankton net during night flood tides from the north end of the Yaquina Bay Fishing Pier (44.623°N, −124.055°W) on 6/19/2021 (58‐day experiment) and 8/1/2021 (17‐day experiment). The pier extends into the Yaquina Bay entrance channel where it is in the direct path of incoming nearshore coastal water. We distinguished *O. griffenis* cryptoniscans from the only other common Yaquina Bay cryptoniscan species, *Ione cornuta* (Bate, 1865), by the longer uropods and subchelate pereopods of *I. cornuta* relative to the short uropods and simple pereopods of *O. griffenis*. The two experiments were initiated within 48 and 24 h of the plankton collections.

Cryptoniscans for all treatments were held in covered 50 mm diameter glass Petri dishes of seawater. For the 58‐day experiment, we divided 30 cryptoniscans among six Petri dishes of five specimens each and maintained two replicates in each treatment condition (Table 1). A cryptoniscan in poor condition in a 20°C dark replicate on the first day of the 58‐day experiment was removed without replacement, and thus, the total initial cryptoniscans in the 58‐day survival test were reduced to 29. The non‐aerated seawater in each replicate was exchanged at approximately two‐day intervals during mortality inspections. Cryptoniscans were inspected and counted under 6× magnification. Specimens unresponsive to probing were classified as dead and removed without replacement. Cryptoniscan survival remained significant at day 41 of the 58‐day test. Regular monitoring was discontinued for the subsequent days due to remote fieldwork. The 17‐day experiment (unexchanged ballast water survival experiment) consisted of 33 cryptoniscans distributed among the three experimental treatment conditions in single replicates of 11 specimens each.

**TABLE 1:** *Orthione griffenis* cryptoniscan larval mortality, half‐life, and estimated drift distance under two experiments (58‐day, 17‐day) with three treatment conditions (12°C dark, 20°C dark, 20°C diel cycle).

	Treatment condition	Mortality	Half‐life (days)	Drift distance (km)
58‐day	12°C dark (*n* = 5)	0.026	26	1300
	12°C dark (*n* = 5)	0.004	166	8300
	20°C dark (*n* = 5)	0.045	16	800
	20°C dark (*n* = 5)	0.028	24	1200
	20°C diel (*n* = 5)	0.031	23	1050
	20°C diel (*n* = 4)	0.017	40	2000
	Pooled (*n* = 29)	0.022 (0.014–0.034)	31 (20–49)	1550 (1000–2450)
17‐day	12°C dark (*n* = 11)	0.012	60	3000
	20°C dark (*n* = 11)	0.006	124	6200
	20°C diel (*n* = 11)	0.012	60	3000
	Pooled (*n* = 33)	0.010 (0.001–0.018)	72 (38–562)	3600 (19900–28,100)

*Note:* The numbers of initial cryptoniscans are shown in parentheses after each treatment condition. For the pooled results, the 95% confidence intervals are shown in parentheses.

Mortality rates were calculated from 22 observations for the 58‐day experiment and 2 from the 17‐day experiment (Table 1). The mortality rate was estimated as the ratio of deaths to exposure in the experimental period, where exposure is defined as the total survival time of all observed organisms combined. For example, if two cryptoniscans survive for 7 days each and 3 cryptoniscans survive for 10 days each, the 7‐day and 10‐day survivors contribute 14 and 30 days of exposure, respectively, and the mortality rate is 5/44. This is the maximum likelihood estimator of the mortality rate assuming a constant hazard (Andersen et al. 1991). A pooled mortality rate was also calculated over all treatment conditions for the 58‐day and 17‐day experiments, respectively, and a 95% confidence interval was derived by assuming a Poisson‐distributed death count.

We created an index of dispersal potential, **D**, for *Orthione* cryptoniscans by calculating the maximum distance a large population might travel before 50% dies out. To do this, we first estimated population half‐life days, ***H***, from the 58‐day and 17‐day experiments. When the surviving population is 50% of the initial population, 0.5 = e^bH^, where b is the mortality rate. Therefore:

(1) $$H=-\ln\left(2\right)/b$$

***D*** can then be calculated as

(2) D=HV

where ***V*** is the prevailing coastal current velocity.

*Orthione griffenis* cryptoniscans are present year‐round and abundant in Yaquina Bay and, presumably, also in coastal nearshore waters at least from March through August (Chapman et al. 2012). We therefore used Behrens‐Yamada et al.'s (2021) summarized sea surface temperatures and prevailing current velocity, ***V***, along the northeast Pacific coast for our comparisons of larval dispersal. The Davidson current can exceed 50 km per day from September to April during strong El Niño events (Austin and Barth 2002; Huyer et al. 1998; Behrens Yamada et al. 2021). Assumptions of cryptoniscans as simple propagules are warranted given that they cannot feed and must survive entirely on internal resources until they settle onto their final decapod hosts. While other currents may influence cryptoniscans' dispersal, our analysis focused on northward transport potential from California, the known origin of invasion.

## 3 Results

### 3.1 Sequencing Results

ddRAD sequencing produced a total of 441,654,641 raw reads, with the mean reads per sample being 3,840,475. The average sequencing depth per sample was 33×. The 80% coverage dataset created with STACKS and VCFtools resulted in a total of 926 SNPs.

### 3.2 Population Differentiation and Structures

Overall, H_O_, H_S_, and H_T_ for *Orthione griffenis* were 0.04, 0.32, and 0.33 respectively, demonstrating low to moderate genetic diversity across sampling locations. The inbreeding coefficient F_IS_ was 0.89, suggesting a low level of divergence in these populations. Tajima's *D* was −3.49 (*p* < 0.001), which indicated a significantly lower‐than‐expected frequency of genetic polymorphisms (Hahn 2018). The average F_ST_ value for the sampled populations was 0.01. Pairwise F_ST_ values were low between most populations (Table 2) and the largest pairwise F_ST_ value (0.05) was between Calvert Island, BC, and Tomales Bay, CA, and between Calvert Island and Sitka, AK; but only the value between Calvert Island and Tomales Bay was significant.

**TABLE 2:** Pairwise Weir and Cockerham's F_ST_ between eight *O. griffenis* sampling locations.

Population	Morro Bay	Tomales Bay	Humboldt Bay	South Slough	Yaquina Bay	Calvert Island	Ketchikan
Tomales Bay	0						
Humboldt Bay	0	0.03*					
South Slough	0	0.03	0				
Yaquina Bay	0	0.01	0.01	0			
Calvert Island	0	0.05*	0.04	0.01	0.03		
Ketchikan	0	0	0.01	0	0	0	
Sitka	0	0.01	0.01	0	0	0.05	0

*Note:* Significant *p* values were indicated with “*”.

**FIGURE 3:**
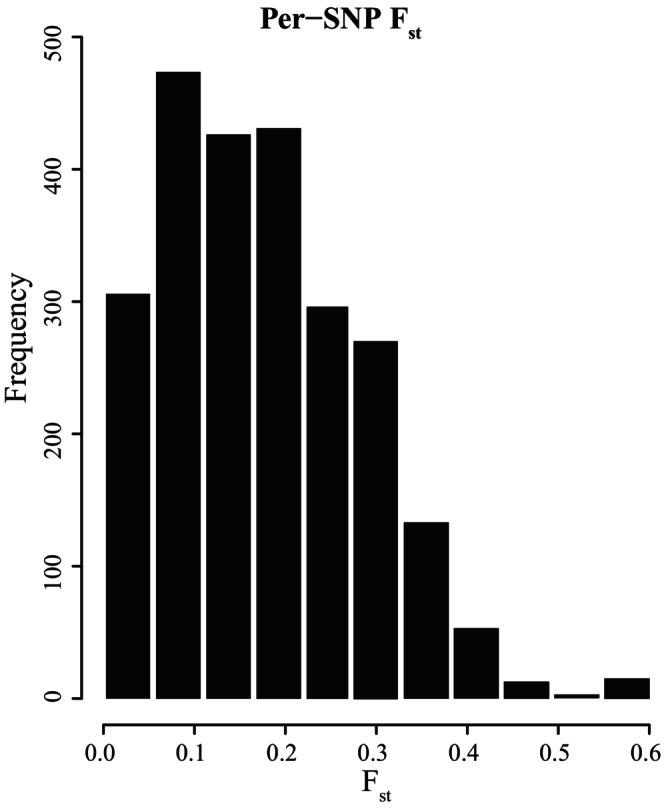
Per‐SNP F_st_ frequencies among *Orthione griffenis* populations.

**FIGURE 4:**
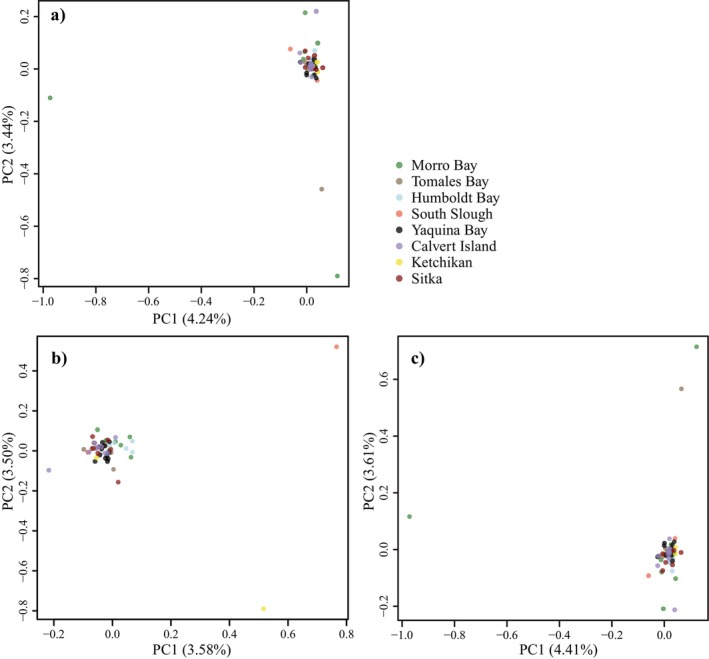
Principal component analyses plots of overall genetic differences among *Orthione giffenis* populations: (a) Includes all 56 sampled individuals, (b) includes 49 individuals after outlier removal, and (c) includes 46 individuals after outlier removal.

**FIGURE 5:**
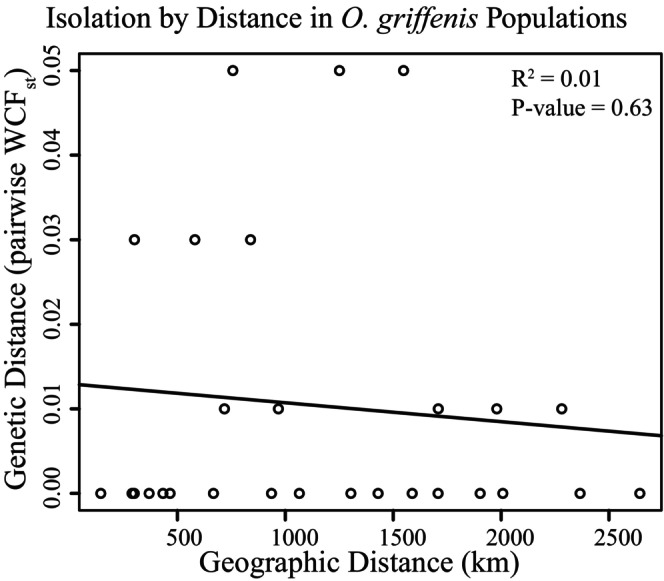
Linear regression measuring the relationship between geographic distance (km) and genetic distance (Weir and Cockerham's *F*_*ST*_) for *O. griffenis* populations.

Measures of F_ST_ on a per‐SNP basis showed that most of the 926 SNPs display little to moderate structure (F_ST_ < 0.25), but a small percentage showed moderate to moderately high structure (Figure 3). Outlier scans did not detect any significant outlier loci. PCA showed that most samples clustered together (Figure 4), further indicating a lack of differentiation between *O. griffenis* populations. After removing outliers, the clustering patterns remained the same. The effect of isolation by distance (Figure 5) was also not significant (*r* ^2^ = −0.03, *p* = 0.63).

**FIGURE 6:**
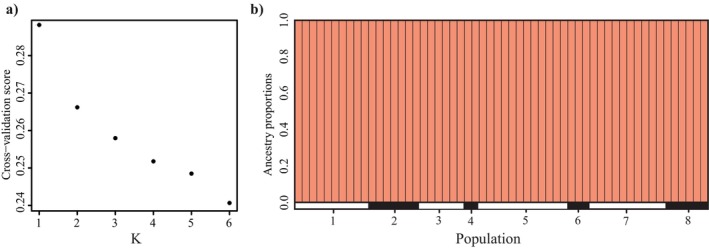
(a) Cross‐validation in TESS is used to determine the most likely *K*‐value by identifying the point where the decrease in cross‐validation score begins to plateau, indicating an optimal balance between model complexity and fit. (b) TESS plots showing estimated ancestry proportions based on a cluster value of 1. Each bar represents an individual with the most southern populations shown on the left and the most northern populations on the right. The numbers corresponding to each population are as follows: 1. Morro Bay, CA, 2. Tomales Bay, CA, 3. Humboldt Bay, CA, 4. South Slough, OR, 5. Yaquina Bay, OR, 6. Calvert Island, BC, 7. Ketchikan, AK, 8. Sitka, AK.

TESS analysis was also used to visualize the potential clustering between populations. *K*‐values from 1 to 3 were used in the analysis, and cross‐validation indicated a *K* value of 1, indicating *O. griffenis* populations consist of one ancestral contribution (Figure 6).

We used AMOVA to test whether there was significant genetic differentiation between *O. griffenis* populations north and south of Cape Mendocino, CA, a suspected location of population differentiation in the *U. pugettensis* host populations (Horan 2021). Most of the genetic variation among *O. griffenis*, however, was partitioned within populations between individuals and within individuals (Table 3). Variation between populations is minor, and there is no significant genetic variation between the ranges north and south of Cape Mendocino, CA (Table 3).

**TABLE 3:** Analysis of molecular variance (AMOVA) to test for northern and southern range subdivision in *Orthione griffenis*.

Source of variation	d.f.	% variation	*p*
Between range	1	0.46	0.19
Between populations within range	6	2.57	0.18
Within populations	48	85.68	0.01*
Within individuals	56	11.29	0.01*

*Note:* Significant *p* values were indicated with “*”.

### 3.3 Average Cryptoniscan Survival Times

The calculated larval mortality rates (Table 1) were low across all experimental conditions and appeared to be the highest in a 20°C dark treatment and the lowest in a 12°C dark treatment of the 58‐day experiment. Overall mortality was 0.022 in the 58‐day experiment and 0.010 in the 17‐day experiment. Individual half‐life estimates mostly fell within 16–60 days, except for an outlier in the 58‐day experiment (166 days) and one in the 17‐day experiment (124 days). The overall half‐life was 31 days with 95% confidence intervals of 20–49 days in the 58‐day experiment. The overall half‐life of the 17‐day experiment was 72 days, and because of the smaller sample size, the 95% confidence interval was much wider (Table 1).

Using the more reliable 58‐day overall half‐life estimation, a passive cryptoniscan in the Davidson Current (50 km/day) could disperse between 1000 to 2450 km under various temperature and light conditions. For perspective, the earliest record of *O*. *griffenis* in North America is 1988, from Willapa Bay, WA, which is approximately 1400 km south of Sitka, AK, the most northern known population of *O. griffenis* (Figure 2).

## 4 Discussion

### 4.1 Inferred *Orthione Griffenis* Invasion History

*Orthione griffenis* appears to have established and invaded North America from Morro Bay, California, to Sitka, Alaska, from a single founding population that dispersed to its present range in long‐shore coastal currents over several decades. Genetic analyses revealed a lack of structuring among *O. griffenis* across this range—a striking yet recurrent pattern observed in other northern Pacific marine invasions (Tepolt et al. 2009, Le Cam et al. 2020). The low overall F_ST_ and high F_IS_ indicated panmixia among populations. Tajima's D values are consistent with patterns of population expansion after a bottleneck event (Hahn 2018). The lack of distinct PCA clusters or variations explained by geographic factors was also consistent with low population divergence. Additionally, TESS results indicate that a single ancestry contribution is most likely within these populations.

The lack of genetic diversity coupled with long‐range larval dispersal in northeastern Pacific *O. griffenis* is consistent with a single invasion stemming from a founder population, which is common in biological invasions (Allendorf and Lundquist 2003; Eckert et al. 2008; Therriault et al. 2005). Chapman et al. (2012, 2021) proposed that San Francisco Bay is the most likely initial receiving harbor of *O. griffenis* based on first discoveries among major trans‐Pacific shipping ports (San Francisco Bay, Long Beach/Los Angeles Harbor, or The Salish Sea). San Francisco Bay is also the likely initial point of establishment for a large fraction or majority of all introduced northeastern Pacific marine species (Cohen and Carlton 1998). The prevailing winter–spring north‐flowing Davidson Current occurs when larval *O. griffenis* could be transported from San Francisco Bay to the Strait of Juan de Fuca, Washington, and the Salish Sea. The south‐flowing summer‐to‐fall California Current could transport larvae from San Francisco Bay to Morro Bay, an early invasion site that is the southern range of *Upogebia pugettensis* (Chapman et al. 2012). Our larval dispersal distance estimations also support these possibilities. Nevertheless, the potential of multiple *O. griffenis* invasions in the Northeast Pacific cannot be ruled out entirely. For instance, genetically homogeneous O. griffenis populations may have been introduced from Asia multiple times. Alternatively, genetic homogeneity could result from genetic drift or the rapid mixing of small, initially divergent populations.

Asia is the most prolific source of marine species introductions to western North America (Cohen and Carlton 1998; Ruiz et al. 2000; Blakeslee et al. 2013). The bopyrid isopod species diversity of East Asia is at least 10 times greater than that in North America (Chapman et al. 2012). Our 17‐day larval survival experiment indicates that *O. griffenis* are competent to survive in trans‐Pacific trips in ballast water traffic. Isolating precise original sources of invasion will require information on the genetic makeup of Asian *O. griffenis* populations. Comparative analyses between North American and Asian populations will provide opportunities to isolate the most likely mechanisms of *O. griffenis* trans‐Pacific introduction.

### 4.2 Additional Host Species on the West Coast

This study only included *O. griffenis* associated with *U. pugettensis* . In estuaries south of Morro Bay and Point Conception (Figure 2), California, *O. griffenis* infests a different native host, *Upogebia macginitieorum* (Williams 1986). The ecology and impact of *O. griffenis* in those ecosystems remain unresolved. For instance, *O. griffenis* is unlikely to have been established in southern California by passive drift from the northern populations. The prevailing nearshore winter and spring currents of the Los Angeles Bight flow from south to north and converge at Point Conception with the north‐to‐south flowing California Current (e.g., Hickey et al. 1991). Those converging currents thus block direct north or south dispersal in the area. Alternative establishment mechanisms would be dispersal on unknown intermediate copepod hosts or with human‐borne introduction vectors between the north and south. Additional sampling and genetic data are thus needed to resolve whether the southern California *O. griffenis* population is established from northern populations or is from a genetically distinct invading population uniquely adapted to southern California ecosystems. *Orthione griffenis* also infests its original Asian host *Upogebia major* (DeHaan 1841), which was introduced into the San Francisco Bay area in the mid‐2000s (Chapman et al. 2021). Further genetic analyses could reveal whether host species can drive *O. griffenis* divergence and whether *O. griffenis* could potentially threaten more native North American mud shrimp species.

### 4.3 Potential Signs of Population Divergence

The approximately 40‐year span since *O. griffenis* was established is short in evolutionary time, but intense selection on these new northeast Pacific populations could be accelerating their genetic diversification despite the presently observed low population differentiation. The founding *O. griffenis* population is unlikely to have transferred a full complement of Asian genetic diversity to North America, and selection on the North American populations may be ongoing and readily detectable.

Despite the overall low levels of population differentiation, there may be signs of initial divergence among populations. For example, although the majority of the genome did not show large differentiation and there were no significant SNP outliers, the per‐SNP F_ST_ distribution (Figure 3) revealed a small number of loci that are moderately differentiated among individuals; these loci represent potentially divergent sites within the genome. The driving forces of these potential initial divergences are unclear. Future studies focusing on the functional aspects of the more differentiated SNPs may provide more insights into potential divergence in *O. griffenis*. For instance, the European green crab, *Carcinus maenus* (Linnaeus 1758), invasion also occurred within the last 40 years (Behrens Yamada et al. 2021, 2022). Despite a low overall genetic diversity, its populations exhibit differentiated SNPs and a chromosomal inversion associated with an adaptive cline of increasing latitude and decreasing winter temperatures (Tepolt et al. 2021).

### 4.4 Paradox of “Successful” Invasions

The lack of genetic structure in invasive species is not unique to *O. griffenis*. Another major northeast Pacific estuary invader is the gammaridean amphipod, *Grandidierella japonica* (Stephensen 1938), native to northeast Asia. *Orthione griffenis*, *C. maenas*, and *G. japonica* are among the most widely distributed estuary species of the eastern Pacific and have similar low genetic diversity where they cooccur. Examples in other regions include the warty comb jelly, *Mnemiopsis leidyi* (Agassiz 1865), which has lost genetic diversity as it was introduced from the northwest Atlantic into the Black Sea and then from the Black Sea population into the western Mediterranean (Jaspers et al. 2021). The coastal marine brown seaweed, *Sargassum muticum* (Fensholt 1955), is a “worldwide” invader and has almost no genome‐wide genetic variation over most of its circumglobal introduction range following severe founder events (Le Cam et al. 2020).

Many “successful” invasions represent a paradoxical phenomenon where resilience and adaptation to novel environments do not coincide with high genetic diversity. Typically, the resilience of native species is inferred from their continuous geographical ranges and recoveries from ecological stresses (Caswell 2001; Bell 2017) and is generally assumed to increase with genetic diversity (Ehlers et al. 2008). Relations between genetic diversity and species persistence, however, are difficult to measure among introduced populations. Selective mortality in the invasion process, stochastic population dynamics during early establishment, and initial genetic bottlenecks can winnow the genetic diversity of introduced species (Cristescu et al. 2001; Bors et al. 2019). The success of invasive taxa may therefore stem from a few genetic variants of large effect that can compensate for relatively low overall diversity (Tepolt et al. 2021). Invading species may also benefit from losses of maladaptive genetic diversity that was previously adaptive in their donor regions (van't Hof et al. 2016). Currently, data on the traits associated with *O. griffenis* success are limited. Greater knowledge of these processes and mechanisms is needed for mitigating or reversing the effects of established invading species like *O. griffenis*.

### 4.5 Conservation Implications

The rapid *O. griffenis* invasion and consequent depletion of *Upogebia pugettensis* host populations across the Northeast Pacific are likely to be forcing ecological and economic repercussions. All known *U. pugettensis* populations in California, Oregon, and Washington infested by *O. griffenis* have collapsed or are extinct (Chapman et al. 2012, 2021; Chapman and Carter 2014). As of August 2021, *O. griffenis* has spread as far North as Ketchikan and Sitka, AK, where infestation intensities are up to 80%, and functional levels of *U. pugettensis* reproduction are unlikely. Declining mud shrimp populations reduce the number of burrows they make, resulting in a decline in surface areas of tidal flats and habitat availability for native commensal estuarine organisms. Shorebirds and juvenile salmon that rely on these shrimps and their larvae as staple prey sources could be stressed by the loss.

Biological invasions of coastal marine communities have increased dramatically over the last two centuries (Ruiz et al. 2000; Pyšek et al. 2020), and possibly 10% of all shallow water marine species within the Oregonian province of the northeast Pacific are non‐native (Cohen and Carlton 1998; Carlton 2007). The management of international ballast water traffic, ship fouling, live seafood, and pet trade, as well as long‐term ecosystem monitoring, has not been sufficient to stop marine invasions from spreading (Pyšek et al. 2020). Human‐mediated species invasions are increasing with the globalization of ballast water, aquaculture, and the live seafood traffic (Ruiz et al. 2000; Bax et al. 2003; Pyšek et al. 2020) and are only likely to increase with climate change effects (Stachowicz et al. 2002; Solomon et al. 2007). Responses to established invasions are needed both to assess whether prevention efforts are sufficient and also to adapt, control, or mitigate their effects when they are established.

## Author Contributions

**Emily R. Curcio:** conceptualization (equal), data curation (lead), formal analysis (lead), investigation (lead), methodology (equal), project administration (supporting), validation (equal), visualization (equal), writing – original draft (lead), writing – review and editing (equal). **Viridiana Avila‐Magaña:** data curation (equal), formal analysis (equal), software (equal). **Kelly R. Martin:** formal analysis (supporting), methodology (supporting), validation (equal), writing – review and editing (equal). **John W. Chapman:** conceptualization (equal), data curation (equal), formal analysis (equal), funding acquisition (lead), investigation (equal), methodology (supporting), project administration (supporting), resources (supporting), writing – review and editing (equal). **Leanne E. Elder:** conceptualization (equal), funding acquisition (supporting), project administration (equal), resources (equal), supervision (equal), writing – review and editing (equal). **Joshua Mayo:** conceptualization (supporting), data curation (equal), investigation (supporting). **Grace K. Roa:** investigation (supporting), methodology (supporting). **Jingchun Li:** conceptualization (equal), data curation (equal), formal analysis (equal), funding acquisition (lead), investigation (equal), methodology (equal), project administration (equal), resources (equal), supervision (lead), validation (equal), visualization (supporting), writing – original draft (supporting), writing – review and editing (lead).

## Conflicts of Interest

The authors declare no conflicts of interest.

## Supporting information


**Table S1.** Metadata for specimens used in the analyses, including species names, sex, collection data, and voucher IDs.

## Data Availability

The raw sequence data that support the findings of this study are openly available in NCBI Genbank at https://www.ncbi.nlm.nih.gov/sra/PRJNA1073485, PRJNA1073485.
